# Acute Effects of Rest Redistribution Training on Physical and Physiological Responses in Anxious Female College Students

**DOI:** 10.3390/life15040555

**Published:** 2025-03-28

**Authors:** Weihao Cheng, Ran Li, Runsheng Yan, Ruoya Liu, Zeyu Gao

**Affiliations:** 1 School of Strength and Conditioning Training, Beijing Sport University, Beijing 100084, China; chengweihao0907@163.com; 2 School of Sport and Science, Beijing Sport University, Beijing 100084, China; yanrs007@163.com (R.Y.); 2022210380@bsu.edu.cn (R.L.); 3 Dezhou Education Bureau, Dezhou 252000, China; dzsgzy@163.com

**Keywords:** rest redistribution, anxious female college students, countermovement jump, heart rate variability, rating of perceived exertion

## Abstract

(1) Background: This study compares the immediate effects of Rest Redistribution Training (RR) and Traditional Set Structure Training (TS) on vertical jump performance, heart rate variability (HRV), and perceived exertion (RPE) in anxious female college students. (2) Methods: In a randomized experimental design, 14 anxious female college students (ages 18–25, screened via Zung’s Self-Rating Anxiety Scale (SAS) with scores ≥50) underwent a familiarization session followed by two trials involving either a RR or TS conditioning routine. Vertical jump, HRV, and RPE were measured pre- and post-session, and during training, respectively. (3) Results: Both protocols induced significant decrements in squat jump (SJs) and countermovement jump (CMJs) metrics (*p* < 0.05), but no statistically significant between-group differences emerged (*p* > 0.05; SJ height: d = 0.059, 95% CI [−0.05, 0.05]; CMJ peak power: d = 0.253, 95% CI [−0.02, 0.02]). TS induced significant decreases in time-domain HRV indices (SDNN: d = 0.888, 95% CI [1.07, 16.13; RMSSD: d = 1.511, 95% CI [8.87, 27.63]) and high-frequency power (HF: d = 0.788, 95% CI [2.73, 379.71]), whereas RR preserved these indices. RR significantly reduced RPE compared to TS (*p* < 0.05; barbell bench press: d = 1.132, 95% CI [0.28, 1.48]; leg press: d = 0.784, 95% CI [0.01, 1.31]). (4) Conclusions: RR and TS protocols induced comparable decrements in vertical jump performance among untrained anxious female college students under equivalent loads; however, RR demonstrated superior autonomic regulation, reduced perceived fatigue, and equivalent performance outcomes, highlighting its potential as a low-stress alternative to traditional resistance training for anxiety-prone populations.

## 1. Introduction

Anxiety disorders represent a globally prevalent mental health challenge [1], with epidemiological studies indicating a pooled one-year prevalence of 10.6% and lifetime prevalence of 16.6% worldwide. Notably, these conditions demonstrate significant demographic disparities: women exhibit 1.5−2.3 times higher susceptibility than men (annual prevalence: 5.2−8.7% vs. 2.5−4.1%), particularly among young adults (prevalence: 2.5−9.1%) [1]. A meta-analysis further reveals pronounced gender differences in China, where lifetime prevalence estimates reach 2.85% for males compared to 5.37% for females [2]. Evidence suggests that sex-specific neuroendocrine mechanisms, particularly estrogen-mediated modulation of hypothalamic–pituitary–adrenal (HPA) axis reactivity, may underlie these disparities [3]. Anxiety can lead to autonomic nervous system (ANS) disorders, manifested by lower-than-normal heart rate variability (HRV) [4,5]. HRV is widely used to assess ANS modulation. Exercise can improve anxiety, as well as effectively enhance physical health [6,7]. Studies have shown that both aerobic exercise and resistance training can improve HRV [8,9]. Long-term resistance training has been proven to significantly enhance HRV and optimize ANS function among anxious female college students [10]. However, it is important to note that acute resistance training increases cortisol release via HPA axis activation and elevates heart rate (HR) while reducing high-frequency power (HF) through sympathetic excitation [11]; additionally, it transiently suppresses parasympathetic activity, leading to a temporary decrease in HRV [12,13].

The design of a resistance training (RT) program significantly influences training adaptations. Modifications to an RT program—such as the type of muscular contraction, load, volume, rest periods, lift velocity, exercise order, exercise type, and training frequency—can affect its impact on the body [14,15]. Traditionally, repetitions within a set are performed consecutively without rest, followed by a specified inter-set rest period. This is known as the ‘traditional set structure training’ (TS). During a traditional set of RT, individuals typically reach or come near to muscle failure, resulting in physiological fatigue. This fatigue is characterized by decreased concentric velocity and power performance [16], disruption of movement technique [17], accumulation of metabolites [18], decreased cardiac parasympathetic modulation [13], and increased effort perception [19]. However, lower movement velocity and power during acute resistance training may hinder long-term power development [20]. Higher effort perception may also discourage resistance training adherence among the general public seeking health improvements [21]. Notably, resistance training that leads to a reduction in cardiac parasympathetic modulation could potentially elevate the risk of cardiovascular dysfunction [13].

Rest Redistribution Training (RR) has emerged as a viable alternative to mitigate these limitations. This paradigm modifies traditional set configurations by strategically partitioning total rest duration into brief intra-set recovery intervals (e.g., 30 s pauses after every 4 repetitions) while maintaining equivalent aggregate rest time compared to conventional inter-set protocols [22]. Previous research indicates that RR attenuates velocity and power decrements during and post-resistance training while reducing lactate accumulation [23] and lowering ratings of perceived exertion (RPE) [24]. Additionally, RR exerts less stress on the autonomic nervous system, resulting in lower parasympathetic inhibition [25]. With low parasympathetic tone at baseline in anxious individuals [26], TS further inhibits vagal nerve activity due to continuous myofascial pressure, while RR, through intermittent offloading of pressure, may alleviate autonomic stress by reducing the afferent impulse of muscle fusions and reducing sympathetic overactivation. However, the participants in these studies were healthy individuals without anxiety symptoms. Despite evidence supporting RR training in general populations, its effects on neuromuscular function and autonomic response in untrained anxious individuals remain unexplored. Understanding the similarities and differences in the effects of RR and TS on countermovement jumps, heart rate variability, and perceived exertion ratings can provide valuable insights for promoting health among individuals with anxiety. Long-term adaptations in muscle strength, hypertrophy, power output, velocity, and endurance are similar between RR and TS [27,28]. However, RR may achieve these adaptations with less fatigue during resistance training, making it an appealing option. Therefore, the objective of this study was to compare the immediate effects of TS and RR on countermovement jumps (CMJs), heart rate variability (HRV), and rating of perceived exertion (RPE) among anxious female college students. This comparison was conducted using a moderate-intensity resistance training program with equal total training volume, intensity, and rest time between groups.

We hypothesized the following: (a) RR and TS will reduce squat jumps (SJs), countermovement jumps (CMJs), and HRV compared to baseline. (b) RR will lead to more minor decrements in SJs, CMJs, and HRV than TS. (c) RR will be associated with lower RPE than TS throughout training sessions.

## 2. Materials and Methods

### 2.1. Participants

The sample size for this self-controlled trial was calculated based on paired design requirements, using RMSSD (a primary HRV index) data from prior studies [29,30]. We aimed to detect a 25% mean difference (δ = 10 ms) with a standard deviation of differences (σ_diff_) = 8 ms, at α = 0.05 and 80% power (β = 0.20). The formula for paired samples was applied: n = [(Z_α/2_ + Z_β_)/δ × σ_diff_]^2^, where Z_α/2_ = 1.96 and Z_β_ = 0.84. This yielded n ≈ 13. To account for sample attrition, the sample size was increased by 10%, resulting in a minimum of 14 subjects per group.

A total of 14 anxious female college students (18−25 years old) from non-training majors in Beijing Sport University were recruited through social media and personal invitations using the non-probabilistic sampling method (age: 22.6 ± 2.3 years; body mass: 57.4 ± 8.1 kg; stature: 163.2 ± 4.8 cm; body mass index: 21.5 ± 2.7 kg/m^2^). The inclusion criteria were (1) Self-Rating Anxiety Scale (SAS) score ≥ 50 (The mean SAS score of the sample was 57.1 ± 5.1 (range: 51.25−66.25), (2) no cardiovascular diseases or other contraindications to exercise, and (3) no habitual exercise. The experiment received approval from the Ethics Committee of Beijing Sport University and adhered to the principles outlined in the latest revised Declaration of Helsinki (7th revision, October 2013). All participants volunteered to participate in the study. Before the study commenced, they fully understood its content and process and signed the informed consent form.

### 2.2. Study Design

**Figure 1:**
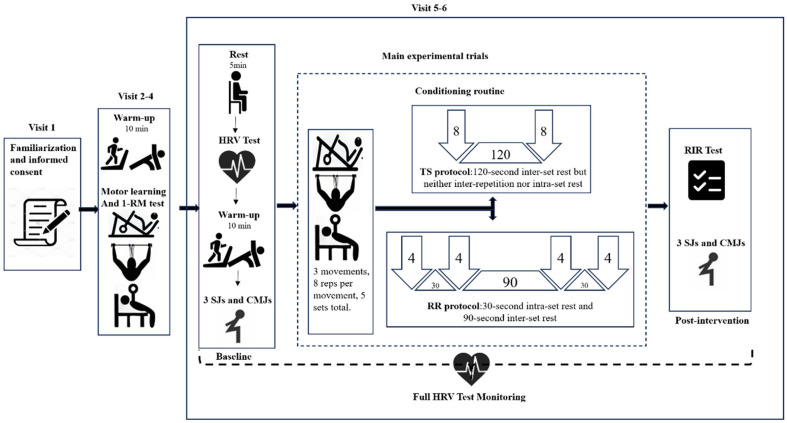
Experimental overview. Visit 1−6 refers to the number of visits to the laboratory.

This study utilized a self-controlled design with randomized cross-over allocation. Participants underwent two experimental trials (RR and TS) in a randomized order, determined by a computer-generated sequence (block randomization, block size = 4). A 7-day washout period was implemented, consistent with literature indicating recovery of autonomic function within 3−5 days [31,32]. Outcome assessors were blinded to the intervention sequence to minimize bias. Participants initially completed a familiarization session, followed by two experimental trials, each separated by 7 days and conducted at the same time of day (±1 h) to ensure consistency. The sample consisted of untrained female college students from Beijing Sport University. This study involved six laboratory visits. The first visit was designed to familiarize participants with the procedures and to obtain their informed consent. Visits two through four were dedicated to motor learning, with 72 h between each visit. Participants’ 1RM data for leg press, lat pulldown, and barbell bench press were collected during Visit four to serve as load references for the formal experiment. Visits five and six were designated for carrying out the experimental conditions. A 7-day washout period separated these sessions. Vertical jumps and HRV were measured before and immediately after each experimental session. The rating of perceived exertion (RPE) was assessed during each training session. Participants were instructed to avoid intense physical exercise and alcohol intake for 48 h, avoid caffeine and alcohol intake for 24 h, and fast for two hours before each laboratory visit. Importantly, all procedures were conducted during the same time window (3 p.m. to 6 p.m.) to avoid performance variations due to testing at different times. The experimental procedure can be referred to Figure 1

### 2.3. Training Protocol

Visits one through four consisted of familiarization sessions aimed at gathering biometric data, explaining the testing procedures, facilitating motor learning, and enabling participants to complete a one-repetition maximum (1RM) test, which included leg press, lat pulldown, and barbell bench press. Upon their first visit to the laboratory, participants had their height and weight measured, and the testing procedures were explained. During the second through fourth visits, participants underwent a 10-min standardized warm-up consisting of 5 min of jogging and 5 min of dynamic stretching. Afterwards, testers instructed participants in the basic movements. A 1RM test for the leg press, lat pulldown, and barbell bench press was conducted after the final movement session. During the fifth and sixth visits, participants arrived at the laboratory and remained seated for 5 min before being fitted with a Polar heart rate monitor and belt for heart rate variability (HRV) testing. The vertical jump test, including both squat jump (SJ) and countermovement jump (CMJ), followed the HRV test. Following the vertical jump test, participants performed training under one of the experimental conditions. Both training regimens consisted of three movements: leg press, lat pulldown, and barbell bench press. The protocols are shown in Table 1. The RR protocol involved 8 repetitions of each movement, with a 30 s intra-set rest after the 4th repetition, a 90-s inter-set rest, and 5 sets of each movement. The TS protocol consisted of 8 consecutive repetitions of each movement without intra-set rest, a 120 s rest between sets, and 5 sets of each movement. All repetitions were executed under direct supervision of research staff to ensure strict adherence to standardized movement tempo. A metronome-regulated cadence (60 beats per minute) was implemented, requiring participants to complete concentric phases within 1 s and eccentric phases over 2 s [33], maintaining controlled velocity throughout the range of motion. Participants’ RPEs were recorded using the repetitions in reserve-based rating of perceived exertion (RIR) scale for resistance training [34] after the completion of each set. SJ and CMJ tests were conducted after the completion of all training, followed by the HRV test.

### 2.4. Measurements

#### 2.4.1. Vertical Jump Testing

Vertical jump testing, including the squat jump (SJ) and countermovement jump (CMJ), was conducted using the EzeJump System (model S-300, Swift Performance Corporation, Brisbane, Queensland, Australia), to evaluate vertical jump performance. At each vertical jump assessment time point, participants completed three repetitions each of SJs and CMJs, with 30 s of rest between attempts. The average performance of SJs and CMJs from the three trials was recorded for subsequent analysis.

Jump height was determined based on flight time for both SJs and CMJs. For the SJ, participants maintained a starting knee angle of 80–100° for at least one second before jumping. In the CMJ, participants selected their own countermovement depth to achieve maximum jump height. Participants kept their hands on their hips throughout the jump to eliminate the influence of arm swing. Two experienced investigators, familiar with jumping diagnostics, monitored the movement execution. Flight time was measured as the duration between take-off and landing, with the assumption of uniform acceleration. Peak power output was calculated using the impulse–momentum approach, following methods from previous research [35]. The magnitude of percentage potentiation was quantified using the following equation: Percent potentiation (%) = [(post-conditioning − pre-conditioning)/pre-conditioning] × 100%. Values less than 100 indicate performance decrements, while values greater than 100 indicate performance improvements [36].

#### 2.4.2. HRV Indicator Evaluation

Participants visited our laboratory, having refrained from any physical activity since waking up, following study pre-conditions: (1) an unchanged sleep pattern the night before; (2) abstinence from alcohol, drugs, or stimulants, including coffee and other stimulants for 24 h prior; (3) refrain from moderate-intensity physical activity for 24 h and vigorous-intensity physical activity for 48 h before the test. After the subjects arrived at the test site, they sat in comfortable chairs and rested for 5 min; they then wore a heart rate meter on their left hand and a heart rate belt around the xiphoid process of the sternum and recorded R-R interval signals for 10 min in a quiet environment under thermo-neutral conditions (22−24 °C and 40–60% relative humidity). The Polar heart rate watch (V800, Finland) and the Polar heart rate band (H10, Finland) were used for collecting R-R interval signals. Kubios HRV Standard 3.4 software (University of Eastern Finland, Kuopio, Finland) was utilized to analyze heart rate variability (HRV) data. This software was employed for calculating HRV indicators in both the time domain and frequency domain. Time domain indices include SDNN (standard deviation of all RR intervals, ms) and RMSSD (square root of the sum of the mean differences between adjacent RR intervals, ms). Frequency domain indices include low-frequency power (LF, 0.04−0.15 Hz; sympathetic activity index), high-frequency power (HF, 0.15−0.40 Hz; vagus nerve activity level index), and LF/HF ratio (sympathetic vagus nerve balance index). The corrections to be made on the RR series are displayed on the RR interval axis. When the corrections are applied, detected artefact beats are replaced using cubic spline interpolation [37]. HRV analyses were conducted by the same trained researcher to ensure reproducible and valid data.

#### 2.4.3. Ratings of Perceived Exertion Testing

Repetitions in Reserve-Based Rating of Perceived Exertion Scale for Resistance Training (RIR) [38] is a validated method used to assess the intensity of resistance training, functioning similarly to the Rating of Perceived Exertion (RPE). However, RIR differs from RPE by quantifying intensity based on the number of repetitions left in reserve, rather than subjective ratings, potentially providing more precise outcomes. In this scale, an RPE of 10 corresponds to 0 RIR, an RPE of 9 corresponds to 1 RIR, and so forth. During the formal experiment, the RIR scale was applied immediately after each training set for every exercise to determine the remaining possible repetitions. Fatigue levels were assessed using this scale, and averages were calculated after all five sets of each exercise. Finally, average fatigue levels across all three exercises were calculated.

#### 2.4.4. One-Repetition Maximum Testing

A muscle strength assessment was performed as the foundation for selecting experimental loads. Before the 1RM test, participants completed a standardized warm-up (5 min of dynamic stretching and 2 sets of 8−12 repetitions with 50% of the estimated 1RM load). Certified trainers supervised all sessions to ensure proper technique and safety (use of spotters for barbell exercises). Load progression followed a conservative approach (≤5% increments) to minimize injury risk in untrained individuals. The test comprised three exercises: leg press, lat pulldown, and barbell bench press. The one-repetition maximum (1RM) indirect test was used to assess participants’ muscle strength. Before commencing the test, participants performed 8 to 12 repetitions with light weights to familiarize themselves with the movements and to ensure a thorough warm-up. They then selected a weight that allowed them to complete 3 repetitions. If more than 3 repetitions were completed, participants rested for 2 min before increasing the weight by 5% for the next set. This process was repeated until participants could perform exactly 3 repetitions. The weight lifted and the number of repetitions were recorded and inserted into the formula: 1RM = [lifted weight × (1 + 0.025 × repetitions)] to estimate the 1RM [39].

#### 2.4.5. Measurement of Anxiety Level

Zung’s Self-Rating Anxiety Scale (SAS) was used to measure the degree of anxiety in the subjects. The Chinese version of the SAS has demonstrated good reliability and validity [40]. The SAS consists of 20 questions. Each question is divided into 4 grades according to its severity, with scores of 1, 2, 3, and 4. Higher scores indicate a higher degree of anxiety. The total score was multiplied by 1.25 to convert it into a standard score. A standard SAS score of 50 or higher was classified as indicating anxiety.

### 2.5. Statistical Analysis

A Shapiro–Wilk test was conducted to assess normality, and Levene’s test was used to verify homogeneity of variance. Data are expressed as mean ± standard deviation (SD). Statistical tests were selected based on data distribution: the independent samples *t*-test and paired samples *t*-test were used for between-group and within-group comparisons, respectively, when normality and homogeneity assumptions were met. For non-normally distributed data, the Wilcoxon rank-sum test was applied. Cohen’s d was reported for effect sizes, categorized as small (0.2), moderate (0.5), or large (0.8). All analyses were performed using SPSS (Version 26.0), with statistical significance set at *p* ≤ 0.05.

## 3. Results

### 3.1. Vertical Jump

**Figure 2:**
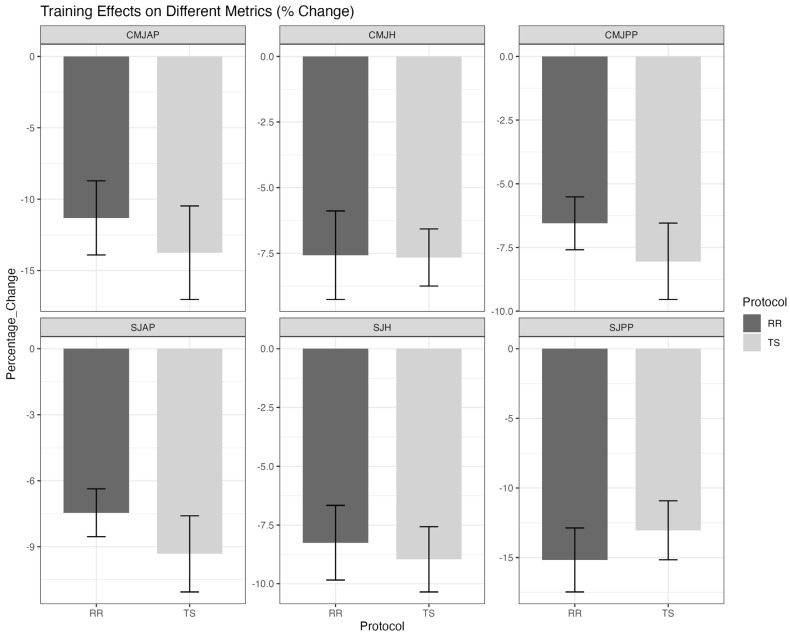
Comparison of SJ and CMJ parameters before and after RR and TS protocol training. TS, traditional set protocol; RR, rest redistribution set protocol; SJH, squat jump height; SJAP, squat jump average power; SJPP, squat jump peak power; CMJH, countermovement jump height; CMJAP, countermovement jump average power; CMJPP, countermovement jump peak power.

The effects on SJs and CMJs are presented in Table 2 and Figure 2. Both protocols induced significant decrements in SJ and CMJ metrics (*p* < 0.05), but no statistically significant between-group differences emerged (*p* > 0.05). Notably, the RR group demonstrated small-to-moderate effect sizes for preserving CMJ peak power (d = 0.253) and height (d = 0.370), suggesting potential practical relevance despite statistical insignificance.

As demonstrated in Table 3, Post hoc power analysis of SJ and CMJ indicators showed that the statistical power of the current sample size (n = 14) was generally less than 25% (range: 6.1~22.7%), far from the standard threshold of 80%.

### 3.2. Heart Rate Variability

**Figure 3:**
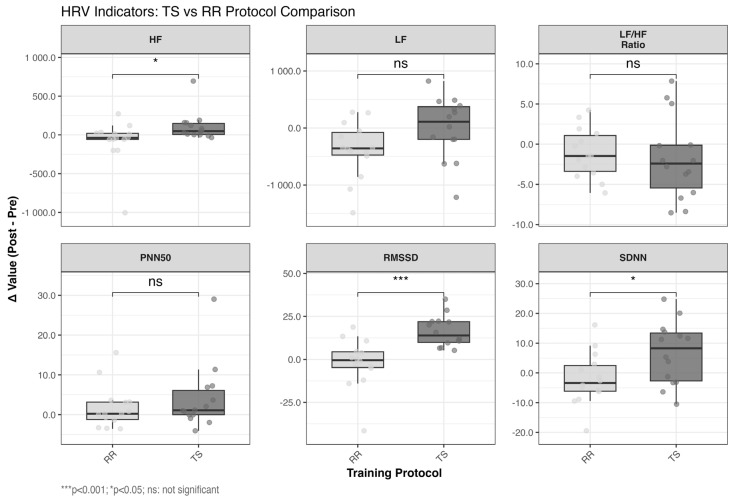
Comparison of the variations in HRV parameters before and after the implementation of RR and TS protocols. TS, traditional set protocol; RR, rest redistribution set protocol; SDNN, standard deviation of all R-R intervals; RMSSD, square root of the sum of the mean of the difference between adjacent RR intervals; LF, low-frequency power; HF, high-frequency power; * Between-group comparison with RR (independent samples *t*-test; *, *p* < 0.05, ***, *p* < 0.01).

The effects on HRV are presented in Table 4 and Figure 3. Specifically, SDNN, RMSSD, and HF in the TS protocol decreased significantly after the experiment (*p* < 0.05), as did LF in the RR protocol (*p* < 0.05). Post-experiment, LF and HF/LF in the TS protocol displayed an increasing trend, while PNN50 showed a decreasing trend. SDNN, LF/HF, and HF in the RR protocol exhibited a decreasing trend, whereas RMSSD and PNN50 showed an increasing trend. However, these changes were not statistically significant compared to pre-experiment levels. When comparing the effects of the two protocols, *TS* induced significant decreases in SDNN (d = 0.888, large effect), RMSSD (d = 1.511, very large effect), and HF (d = 0.788, moderate effect), whereas RR preserved these indices. Between-group comparisons revealed that TS induced significantly greater reductions in SDNN (d = 0.888 vs. d = −0.095), RMSSD (d = 1.511 vs. d = 0.091), and HF (d = 0.788 vs. d = −0.093), indicating robust clinical superiority of RR in autonomic preservation.

### 3.3. Ratings of Perceived Exertion

**Figure 4:**
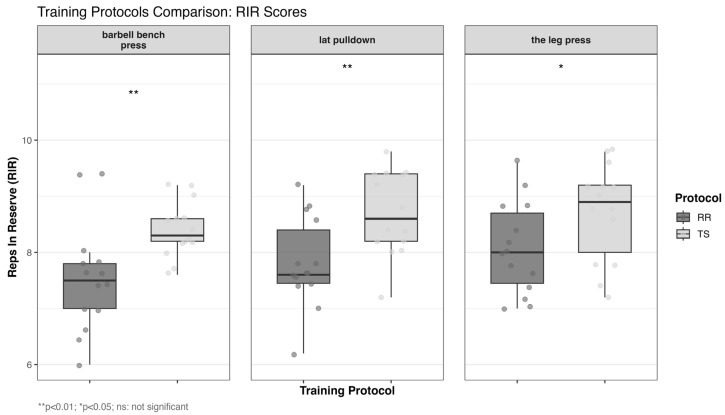
Comparison of RIR parameters: RR and TS protocols training. TS, traditional set protocol; RR, rest redistribution set protocol; * Between-group comparison with RR (independent samples *t*-test; *, *p* < 0.05; **, *p* < 0.01).

The effects on RIR are presented in Table 5 and Figure 4. RR significantly reduced RPE compared to TS across all exercises (*p* < 0.05), with large effect sizes observed in barbell bench press (d = 1.132) and lat pulldown (d = 1.143), and a moderate effect in leg press (d = 0.784). These findings indicate that RR provides substantial reductions in subjective fatigue, which may enhance training adherence.

## 4. Discussion

### 4.1. Comparative Analysis of Vertical Jump Changes

A decrease in vertical jump height is generally considered a reduction in lower limb muscle contraction capacity. Therefore, vertical jump height is also used to evaluate changes in neuromuscular fatigue [41,42]. Additionally, the peak power of the vertical jump can accurately assess the explosive power of the lower limbs [43]. In this study, we selected the leg press, lat pulldown, and barbell bench press as the experimental training movements. These exercises target the primary muscle groups of the upper and lower limbs. Considering that our experimental population consisted of female college students with no training experience, we selected movements suitable for beginners. No additional investigations were conducted on the barbell bench press and lat pulldown movements, as these were only used to induce fatigue and enhance physiological and psychological stimulation during the experimental protocol.

Analysis of vertical jump height and power before and after the experiment revealed a significant decrease in the SJ and CMJ indices for both groups, and both experimental protocols induced significant neuromuscular effort in the subjects. Previous studies indicate that RR can maintain movement speed and power output during training [44,45]. We hypothesized that the vertical jump indices of female college students in the RR group would be significantly better than those in the TS group. Specifically, we expected the rate of change in SJ and CMJ height, average power, and peak power to be lower in the RR group compared to the TS group after the experiment. However, the experimental results showed no significant difference in the rate of change for each vertical jump index between the RR group and the TS group before and after the experiment (*p* > 0.05). This result did not support our hypothesis. Notably, the RR group demonstrated small-to-moderate effect sizes for preserving CMJ peak power (d = 0.253) and height (d = 0.370), suggesting potential practical relevance despite statistical nonsignificance. These trends align with prior research indicating RR’s ability to attenuate power decrements during resistance training [23]. The post hoc power analysis revealed alarmingly low statistical power (6.1−22.7%) for detecting between-group differences (Table 3), falling substantially below the 80% conventional threshold. This elevated Type II error risk suggests that our null findings may reflect inadequate sensitivity rather than true equivalence between protocols.

One reason for the lack of significant differences between the two groups may be that, from a safety perspective, the fatigue induced by the experimental program did not reach the point of exhaustion in subjects with no training experience. This may have prevented the full demonstration of the effects of rest redistribution training on anxious female college students. Future studies could explore whether inducing greater fatigue to the point of exhaustion can better highlight differences between rest redistribution training and traditional resistance training. Another reason could be the small sample size of this study. The sample size calculation was based on the HRV index as the primary outcome, which might not have adequately powered the study to detect significant differences in CMJ indices. Post hoc efficacy analysis showed that 56 to 223 participants were required to express the significance of SJ and CMJ measures. Future research could benefit from an optimized experimental design with a larger sample size to enhance statistical power, potentially yielding more valuable insights into CMJ indices.

In a previous study, Merrigan et al. [45]. examined 12 female undergraduate physical education students who performed deep squats at 70% 1RM, completing four sets of 10 repetitions each. They found that the Rest Redistribution group’s rate of change in movement speed and power was slightly lower than that of the traditional resistance training group (24% vs. 32%). Specifically, movement speed decreased by −7.22% in both groups, while power decreased by −7.87%. Other studies have reported comparable findings: lat pulldown movement resulted in an 18.5% change [46], barbell bench press showed changes ranging from 20% to 23% [47], and leg press demonstrated a 37% change [48]. Additionally, some studies have reported power attenuation of 2−6% and a 3% decrease in movement velocity. Our study showed that no statistically significant differences were found in the rates of change in height and power between the TS and RR groups; comparisons showed that the rates of change in CMJ height (−9.7% ± 6.0% vs. −7.4% ± 3.8%, *p* = 0.320), average CMJ power (−14.4% ± 11.2% vs. −11.5% ± 9.1%, *p* = 0.979), and CMJ peak power (−8.4% ± 5.1% vs. −6.5% ± 3.6%, *p* = 0.366) were lower in the RR group. Although changes in CMJs and SJs did not reach statistical significance, the RR group demonstrated a 2–3% lower reduction in peak power output, which may still be practically relevant for neuromuscular performance. Furthermore, although the between-group comparison of post-experimental changes in CMJ height and peak power did not reach statistical significance, it may be clinically valuable to assess whether these changes meet the threshold for a Minimal Clinically Important Difference (MCID) [49] in practical training.

Another noteworthy point is that the SJ reflects the concentric contraction of the muscles of the lower limbs, as the action of the SJ is to squat with the knees bent to 90°, with the thighs parallel to the ground, and then jump with maximal strength after holding the position for 3 s. CMJs require rapid squatting followed by a maximal jump, during which the extensor muscles of the lower limbs are pre-tensioned for eccentric contraction and then rapidly transition to concentric contraction. This triggers the stretch reflex, and the elastic potential energy of the muscles and tendons is utilized to increase the mechanical power output during the concentric phase. This form of contraction is known as the stretch–shortening cycle (SSC). Typically, the CMJ will have a higher jump height and power output than the SJ [50]. It is generally considered that the utilization of the SSC can result in a performance improvement of up to 11%, which is considered good, and up to 20%, which is considered excellent [51]. Therefore, we believed that due to the presence of SSC, the height and power of CMJs would be higher than that of SJs. However, when comparing the pre-experimental SJ and CMJ heights (RR group: 21.1 ± 2.7 cm vs. 21.4 ± 2.6 cm, TS group: 21.2 ± 2.1 cm vs. 20.9 ± 1.8 cm), average power (RR group: 362.9 ± 137.4 W vs. 347.5 ± 133.4 W, TS group: 344.2 ± 149.8 W vs. 338.2 ± 148.5 W), and peak power (RR group: 1561.0 ± 199.1 W vs. 1516.0 ± 182.6 W, TS group: 1150.4 ± 225.4 W vs. 1489.0 ± 215.9 W), our results indicate that the CMJ indices were not higher than the SJ indices.

This apparent SSC dysfunction in CMJ performance may stem from the following: a) Inexperienced participants demonstrated inadequate pre-activation of triceps surae prior to ground contact [52], compromising elastic energy storage capacity. b) Anxiety-induced parasympathetic suppression [53] potentially disrupted intermuscular coordination, attenuating protocol-specific SSC utilization. c) Non-athletes’ limited motor skill acquisition likely impaired optimal force-time characteristics during the eccentric–concentric transition phase.

### 4.2. Comparative Analysis of Heart Rate Variability Changes

HRV serves as a non-invasive indicator of ANS regulation, reflecting the balance between sympathetic (SNS) and parasympathetic (PNS) activity. Acute resistance training typically suppresses PNS activity, evidenced by reductions in HRV indices such as HF, RMSSD, SDNN, and PNN50, with effects persisting up to 24 h post-exercise [25]. While training variables (e.g., sets, intensity, rest intervals) modulate HRV responses, demographic factors like gender and BMI exhibit negligible influence [51].

Our study showed that the TS protocol elicited significant PNS inhibition, specifically: Time-domain indices: SDNN decreased by 6.7 ± 10.5 ms (*p* < 0.05), RMSSD declined by 7.9 ± 11.5 ms (*p* < 0.01), and PNN50 trended downward (6.6 ± 8.5% → 2.6 ± 2.7%, *p* = 0.360). Frequency-domain indices: HF power decreased by 107.6 ± 141.4 ms^2^ (*p* < 0.05), while LF and LF/HF ratio remained unchanged.

These reductions signify diminished vagal tone and overall ANS regulatory capacity, aligning with prior findings that continuous sets in TS exacerbate metabolic stress and SNS dominance [4,25]. In contrast, the RR protocol preserved PNS activity while facilitating SNS engagement: Time-domain indices: SDNN, RMSSD, and PNN50 showed no significant post-exercise changes. Frequency-domain indices: LF increased by 339.2 ± 511.5 ms^2^ (*p* < 0.05), indicating heightened SNS activity, while HF exhibited a non-significant increase (141.2 ± 113.4 ms^2^ → 152.1 ± 94.7 ms^2^, *p* = 0.736).

The attenuated ANS disruption in RR may stem from the following: (a) Intra-set rest intervals reduce lactate accumulation and metabolic stress, mitigating PNS suppression [54]; (b) Brief pauses may alleviate exercise-induced cardiac vagal inhibition, stabilizing baroreflex sensitivity [54]; (c) Intermittent pressure offloading during intra-set rest reduces afferent feedback from muscle mechanoreceptors, curbing sympathetic overactivation [25].

In terms of clinical implications, the preservation of HF and RMSSD in RR suggests a protective effect on vagal tone, critical for populations with baseline autonomic dysregulation (e.g., anxious individuals) [4]. Enhanced LF in RR reflects adaptive SNS engagement without compromising recovery, aligning with its utility in fatigue management [25] These findings corroborate previous studies demonstrating RR’s superiority in balancing ANS stress during resistance training [51].

### 4.3. Comparative Analysis of Ratings of Perceived Exertion Changes

The RIR scale, a subjective rating of resistance training intensity, is considered a more appropriate fatigue scale for resistance training and correlates well with various objective indicators of fatigue [55]. Research indicates that during multiple sets of intense resistance training, subjective fatigue is positively correlated with the total load, regardless of load intensity and rest between sets [56]. Lins et al. conducted various resistance training exercises at 50% 1RM and 70% 1RM with 14 trained young men, performing 3 sets of 12, 9, and 6 repetitions per set, respectively. Their findings revealed that subjective fatigue ratings were higher at 70% 1RM than at 50% 1RM, consistent at 50% 1RM regardless of the reduced repetitions per set, and remained elevated at 70% 1RM despite the reduction in repetitions per set [57]. This suggests that performing multiple sets of resistance training still elicits an RPE response that corresponds to organismal fatigue, with subjective fatigue ratings increasing as fatigue accumulates. Furthermore, theoretically, during low-intensity resistance training, if the organism has not yet reached fatigue, subjective fatigue scores should remain consistent across all groups, regardless of changes in the number of sets and repetitions. Analysis of RIR scores from barbell bench press, lat pulldown, and deadlift leg raises in both groups revealed an increasing trend in fatigue scores. This suggests that both groups reached a point of locomotor fatigue, with fatigue accumulating as the number of training sets increased.

This study used an intensity of 10RM, with eight repetitions per set. Based on RIR scores, the expected RIR score was 8, indicating that two additional repetitions could have been performed. Previous studies have shown that the blood lactic acid response caused by RR is lower than that of TS [58] and enhances muscle oxygen utilization [59], potentially delaying fatigue perception compared to TS. Novice participants demonstrated limited RIR estimation accuracy compared to trained individuals. While experienced lifters exhibit improved RIR accuracy near failure [55], our untrained cohort displayed inverted trends—overestimating reserve capacity despite submaximal efforts (80% 10RM). This constitutes a critical methodological limitation: novice status confounded RIR-based fatigue assessment. Future studies should integrate objective exhaustion markers (e.g., bar velocity < 0.3 m/s) when investigating untrained populations.

From another perspective, since this study was a controlled experiment with a before-and-after design, the subjects’ consistent bias in estimating their remaining repetitions adds a degree of relative reliability in analyzing the trends and differences between the two groups. Comparing the two groups, rest redistribution training significantly reduced subjective fatigue during training (*p* < 0.05), supporting the hypothesis of this study. Rating of perceived exertion (RPE) and training duration are critical determinants of exercise adherence. For adults with health conditions, prolonged training sessions and high RPE levels are strongly associated with reduced compliance [60,61]. Under comparable training intensities, RR protocols may enhance adherence due to their lower RPE, which minimizes psychological and physical strain during exercise.

Despite comparable performance decrements between protocols, RR demonstrated clinically meaningful advantages, including large effect sizes in autonomic preservation (RMSSD: d = 1.511) and moderate reductions in perceived exertion (RPE: d = 0.784–1.143). These findings support RR as a low-stress alternative for anxiety-prone populations, where even small improvements in recovery may enhance long-term adherence.

### 4.4. Limitations

This study has several limitations that warrant consideration. First, the small sample size (n = 14) resulted in insufficient statistical power (6.1−22.7%) to detect significant differences in vertical jump performance metrics (SJ/CMJ), with post hoc analysis indicating that 56–223 participants would be required to achieve 80% power for these outcomes. Second, the exclusive inclusion of anxiety-naive, untrained female college students limits external validity, as results may not generalize to males, clinical populations, or trained individuals. Additionally, the RIR scale demonstrated novice-related bias, with participants overestimating repetition reserve despite submaximal efforts, introducing uncertainty in fatigue interpretation. Furthermore, the absence of long-term follow-up precludes conclusions about sustained adaptations or clinical translation. Lastly, potential carryover effects from prior sessions may have influenced outcomes in this crossover design, particularly given the high-intensity nature of the protocols. Collectively, these limitations highlight the need for larger-scale studies with objective fatigue markers, longitudinal follow-up, and diverse populations to validate RR’s utility in anxiety management and neuromuscular training contexts.

### 4.5. Future Directions

To address the identified limitations and advance the field, future studies could consider several directions. First, investigating the chronic effects of RR training on strength, power, and autonomic regulation in clinical anxiety populations may provide critical insights into its therapeutic potential. Additionally, exploring sex-specific responses to RR protocols would address gender disparities in anxiety research and optimize exercise prescriptions for males and females. Furthermore, comparing different rest redistribution ratios (e.g., 20 s vs. 40 s intra-set intervals) could identify the optimal balance between performance maintenance and recovery. Moreover, longitudinal designs with objective fatigue markers (e.g., bar velocity, blood lactate) may enhance the validity of fatigue assessment in untrained individuals. Finally, multi-center trials involving diverse demographics would strengthen generalizability and support evidence-based recommendations for integrating RR into anxiety management programs. Collectively, these avenues should refine our understanding of RR’s utility and contribute to personalized exercise strategies for mental health promotion.

## 5. Conclusions

When anxious female college students with no training experience performed the RR and TS protocols, with the same load, the change in vertical jump indices after RR was similar to that of TS. However, the RR protocol showed better autonomic function and lower subjective fatigue, suggesting that RR offers a promising alternative to TS training, reducing autonomic stress and subjective fatigue while maintaining similar performance outcomes.

## Figures and Tables

**Table 1:** Training protocol parameters for RR and TS training.

Parameter	RR Protocol	TS Protocol
Repetitions per set	8 (4 + 30 s intra-set rest + 4)	8 consecutive
Inter-set rest	90 s	120 s
Total sets per exercise	5	5
Total session volume	120 reps (3 exercises)	120 reps (3 exercises)

**Table 2:** Vertical jump performance: pre-test, post-test, and rate of change in SJs and CMJs.

	Protocol	Post-Test/Pre-Test		TS-RR (Between-Group Comparison)				
		Pre-Test	Post-Test	Rate of Change (%)	95%CI	T-Value	*p*-Value	Cohen’s d
		Mean ± SD	Mean ± SD	Mean ± SD				
SJ performances								
Height, cm	TS	21.2 ± 2.1	19.3 ± 2.5 **	−8.6 ± 4.6	−0.05, 0.05	−0.132	0.896	0.059
	RR	21.1 ± 2.7	20.2 ± 2.3 **	−8.6 ± 5.5				
Avg Power, w	TS	344.2 ± 149.8	304.7 ± 144.9 **	−13.1 ± 7.4	−0.05, 0.10	0.683	0.503	0.305
	RR	362.9 ± 137.4	313.4 ± 132.6 **	−15.5 ± 8.0				
Peak Power, w	TS	1150.4 ± 225.4	1391.1 ± 218.3 **	−7.6 ± 5.8	0.00, 0.02	0.136	0.894	0.061
	RR	1561.0 ± 199.1	1445.1 ± 217.3 **	−7.9 ± 5.8				
CMJ performances								
Height, cm	TS	20.9 ± 1.8	19.0 ± 2.0 **	−9.7 ± 6.0	−0.07,0.02	−1.023	0.320	0.457
	RR	21.4 ± 2.6	19.8 ± 2.3 **	−7.4 ± 3.8				
Avg Power, w	TS	338.2 ± 148.5	296.8 ± 142.7 **	−14.4 ± 11.2	−0.12, 0.12	−0.027	0.979	0.012
	RR	347.5 ± 133.4	313.4 ± 132.6 **	−11.5 ± 9.1				
Peak Power, w	TS	1489.0 ± 215.9	1368.0 ± 201.1 **	−8.4 ± 5.1	−0.02, 0.02	−0.927	0.366	0.415
	RR	1516.0 ± 182.6	1416.4 ± 173.5 **	−6.5 ± 3.6				

Note: TS, traditional set protocol; RR, rest redistribution set protocol; SJ, squat jump; CMJ, countermovement jump. * Significantly different from pre-test (paired samples *t*-test; **, *p* < 0.01). Between-group comparison with RR (independent samples *t*-test).

**Table 3:** Post hoc power analysis: effect sizes, statistical power, and recommended sample sizes for vertical jump metrics.

Parameter	Cohen’s d	Current Power	Recommended *n*
SJH, cm	0.375	22.7%	56
SJAP, w	0.063	6.1%	253
SJPP, w	0.245	15.2%	82
CMJH, cm	0.370	22.3%	57
CMJAP, w	0.124	7.8%	179
CMJPP, w	0.253	15.8%	78

Note: SJH, squat jump height; SJAP, squat jump average power; SJPP, squat jump peak power; CMJH, countermovement jump height; CMJAP, countermovement jump average power; CMJPP, countermovement jump peak power.

**Table 4:** HRV indices: pre-training and post-training comparison between RR and TS protocols.

	Protocol	Post-Test/Pre-Test		TS-RR (Between-Group Comparison)				
		Pre-Test	Post-Test	Variation Difference	95%CI	T-Value	*p*-Value	Cohen’s d
		Mean ± SD	Mean ± SD	Mean ± SD				
Time domain								
SDNN, ms	TS	35.2 ± 8.1	28.5 ± 10.0 *	−6.7 ± 10.5	1.07,16.13	2.348	0.027 ^##^	0.888
	RR	31.7 ± 7.1	32.6 ± 5.7	−0.8 ± 7.2				
RMSSD, ms	TS	26.7 ± 10.0	18.8 ± 8.2 **	−7.9 ± 11.5	8.87,27.63	3.999	0.000 ^###^	1.511
	RR	21.3 ± 8.8	20.5 ± 4.4	0.8 ± 9.1				
PNN50, %	TS	6.6 ± 8.5	2.6 ± 2.7	4.24 ± 8.19	−3.07,8.16	0.932	0.360	0.352
	RR	4.3 ± 5.6	2.5 ± 2.2	1.8 ± 5.4				
Frequency domain								
LF, log	TS	550.5 ± 359.1	554.6 ± 489.5	4.1 ± 545.4	−25.25,783.77	1.927	0.065	0.728
	RR	461.3 ± 323.8	800.5 ± 562.6 *	−339.2 ± 511.5				
HF, log	TS	216.4 ± 235.7	108.7 ± 94.3 *	107.6 ± 141.4	2.73,379.71	2.085	0.047 ^#^	0.788
	RR	141.2 ± 113.5	152.1 ± 94.7	−10.9 ± 118.0				
LF/HF	TS	4.5 ± 4.3	6.3 ± 3.7	−1.8 ± 5.1	−3.99,2.58	−0.44	0.664	0.166
	RR	4.8 ± 3.3	6.0 ± 3.9	−1.2 ± 3.1				

Note: TS, traditional set protocol; RR, rest redistribution set protocol; SDNN, standard deviation of all R-R intervals; RMSSD, square root of the sum of the mean of the difference between adjacent RR intervals; LF, low-frequency power; HF, high-frequency power. * Significantly different from pre-test (paired samples *t*-test; *, *p* < 0.05; **, *p* < 0.01). # Between-group comparison with RR (independent samples *t*-test; ^#^, *p* < 0.05; ^##^, *p* < 0.01; ^###^, *p* < 0.001).

**Table 5:** Ratings of perceived exertion: comparison of RIR scores between RR and TS protocols.

Exercise	Protocol	Mean ± SD	95%CI	T-Value	*p*-Value	Cohen’s d
barbell bench press	TS	8.4 ± 0.5	0.28,1.48	2.995	0.006 **	1.132
	RR	7.5 ± 1.0				
lat pulldown	TS	8.7 ± 0.8	0.28,1.49	3.024	0.006 **	1.143
	RR	7.8 ± 0.8				
the leg press	TS	8.7 ± 0.9	0.01,1.31	2.075	0.048 *	0.784
	RR	8.1 ± 0.8				

Note: TS, traditional set protocol; RR, rest redistribution set protocol; * Between-group comparison with RR (independent samples *t*-test: *, *p* < 0.05; **, *p* < 0.01).

## Data Availability

All data generated or analyzed during this study are included in the article.

## Abbreviations

The following abbreviations are used in this manuscript:

RR	Rest Redistribution Training
TS	Traditional Set Structure Training
VJ	Vertical Jump
HRV	Heart Rate Variability
RPE	Perceived Exertion Rating
SJ	Squat Jump
CMJ	Countermovement Jump
SDNN	Standard Deviation of Normal-to-Normal Intervals
RMSSD	Root Mean Square of Successive Differences
ANS	Autonomic Nervous System
RT	Resistance Training
1RM	One-Repetition Maximum
LF	Low-Frequency Power
HF	High-Frequency Power
RIR	Repetitions in Reserve-Based Rating of Perceived Exertion Scale for Resistance Training
SAS	Self-Rating Anxiety Scale
MCID	Minimal Clinically Important Difference
SSC	Stretch-Shortening Cycle
